# Beta-sitosterol-induced Acute Pancreatitis: A Case Report and Review of the Literature

**DOI:** 10.7759/cureus.7407

**Published:** 2020-03-25

**Authors:** Alyssa Lorenze, William Hsueh, John Nasr

**Affiliations:** 1 Pediatrics, West Virginia University School of Medicine, Ruby Memorial Hospital, Morgantown, USA; 2 Gastroenterology, West Virginia University, Morgantown, USA

**Keywords:** pancreatitis, benign prostatic hyperplasia, steroids, phytosterol, herbal supplements, drug-induced pancreatitis

## Abstract

While drug-induced pancreatitis from corticosteroids has been well described in the medical literature, the exact mechanism is unclear. We present the first reported case of drug-induced pancreatitis from beta-sitosterol, a naturally occurring plant sterol structurally similar to cholesterol, obtained primarily through Western diet and supplementation.

A 57-year-old male with a history of situs inversus and benign prostatic hyperplasia presented from an outside facility with a two-day history of worsening epigastric pain radiating to the right upper quadrant. Lipase was markedly elevated at 572 U/L. CT scan and ultrasound of the abdomen were remarkable for acute pancreatitis with acute necrotic collections and normal appearing gallbladder and bile ducts without the presence of gallstones. The patient was managed with aggressive intravenous hydration and supportive management and had resolution of symptoms. At his follow-up appointment, the patient disclosed that he had started a new herbal supplement, beta-sitosterol, on the morning after his symptoms began. Abdominal magnetic resonance cholangiopancreatography obtained at follow-up appointment showed interval resolution of pancreatitis and normal biliary anatomy. In the absence of classical risk factors for acute pancreatitis, a diagnosis of drug-induced pancreatitis secondary to beta-sitosterol was made. The patient was advised to avoid beta-sitosterol, and thus continued to remain asymptomatic.

We describe the first reported case of drug-induced pancreatitis from beta-sitosterol, a common phytosterol found in many over the counter supplements worldwide. After a thorough workup to exclude other causes, our case demonstrates consistent resolution of symptoms and pancreatic enzymes along with normal imaging following discontinuation of the offending agent.

## Introduction

The use of herbal supplements continues to rise as an alternative to conventional medical treatment. Consumers often consider herbal supplements to be safer than standard medical treatment because they are termed “natural” and thus promote a sense of health and well-being under a patient’s control [1,2]. Most patients do not realize however that the Food and Drug Administration (FDA) does not require manufacturers to report the safety and efficacy of these drugs before they are marketed. Likewise, these products often contain unknown impurities and variability in potency of active ingredients. Although the adverse effects and interactions of herbal supplements are poorly understood, a detailed medication history including over the counter and herbal medications is necessary to prevent further drug-induced complications. We report the first probable case of acute pancreatitis secondary to the ingestion of herbal supplementation, beta-sitosterol (B-sitosterol), in the literature thus far.

## Case presentation

A 57-year-old male with a history of situs inversus and benign prostatic hyperplasia (BPH) presented from an outside facility with a two-day history of generalized abdominal pain. The pain was initially characterized as a pressure sensation over his lower abdomen that would come and go. On the morning of admission, the pain became sharp and severe over the midepigastric area, radiating to the right upper quadrant causing the patient to become bedbound. The patient denied a history of smoking, alcohol use, gallstones, recent infection, illicit drug use, trauma, new medications, exposure to scorpions, or weight loss. There was no family history of pancreatic disease. 

**Figure 1:**
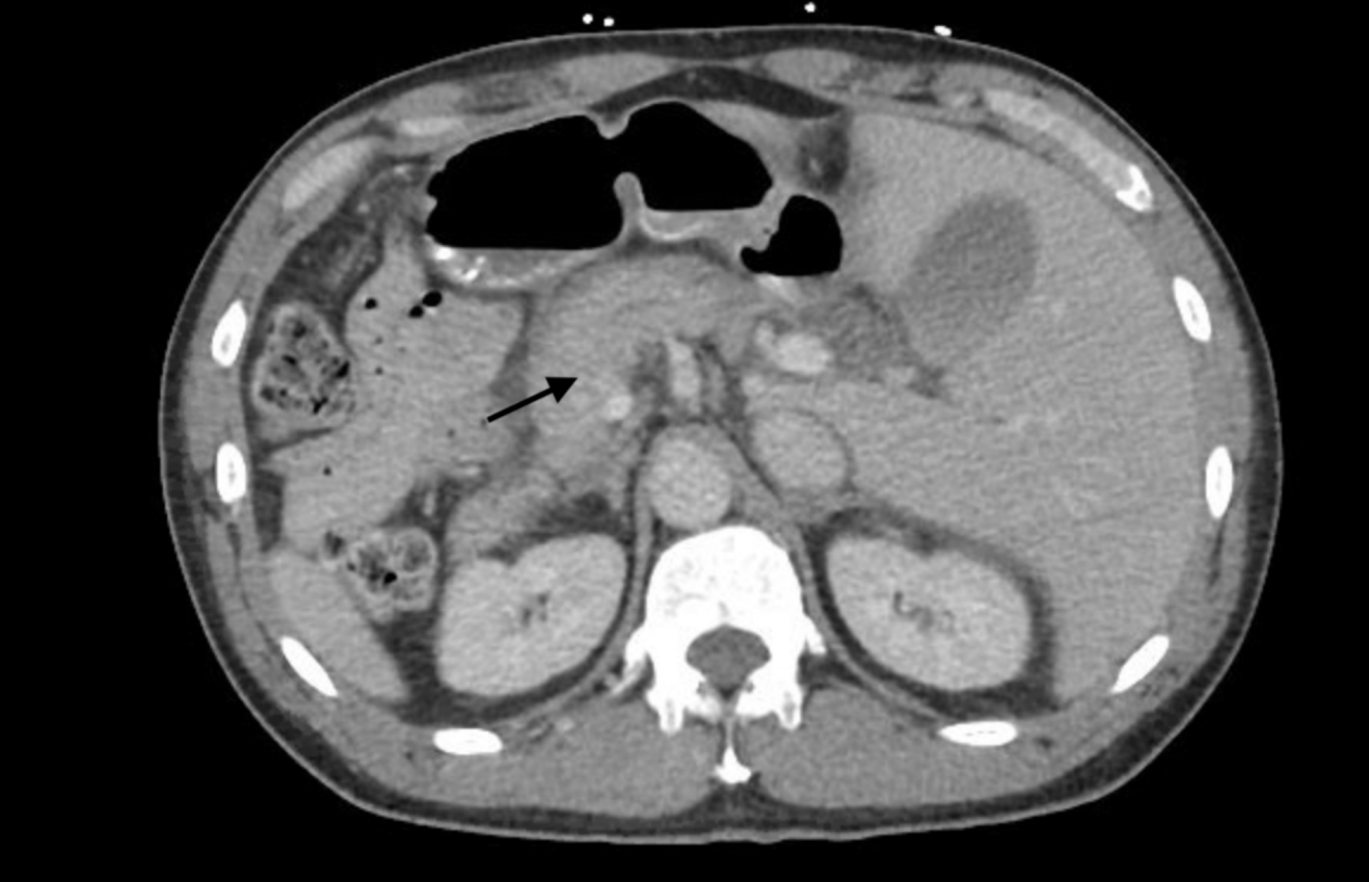
Computed tomography of the abdomen showing heterogenous pancreas with lack of contrast enhancement (arrow)

The patient’s vital signs were within normal limits, and he was afebrile. On examination, the patient was in mild distress with generalized tenderness to palpation over all four abdominal quadrants with localization to the right upper quadrant. He required admission for further investigation and pain control. The patient’s initial laboratory results were noted for a white blood cell count of 10.2, hemoglobin 11.1 g/dl, hematocrit of 31.9%, blood urea nitrogen (BUN) 14 mg/dl, glucose 143 mg/dl, and calcium of 9 mg/dl (normal 8.5-10.2 mg/dl). Liver tests including aspartate aminotransferase, alanine aminotransferase, total bilirubin, and alkaline phosphatase were within normal limits. Lipase was markedly elevated at 572 U/L (>7x upper limit of normal). CT of the abdomen was remarkable for acute pancreatitis with acute necrotic collection and normal appearing gallbladder and bile ducts without gallstones (Figure 1).

An ultrasound of the abdomen showed similar findings to the CT abdomen. The patient was managed with aggressive intravenous hydration with lactated Ringer’s solution, and oral feeding was initially held. Oral feeding was resumed after 24 hours. After 48 hours the patient’s hemoglobin decreased to 8.9 mg/dl and hematocrit 25.5%. Other laboratory investigations including BUN, bicarbonate, calcium, and liver tests remained within normal limits and did not vary much from initial presentation. He received approximately 4 L of lactated Ringer’s solution after 48 hours. The patient had significant resolution of symptoms after four days. The patient was discharged home and referred to gastroenterology clinic. 

**Figure 2:**
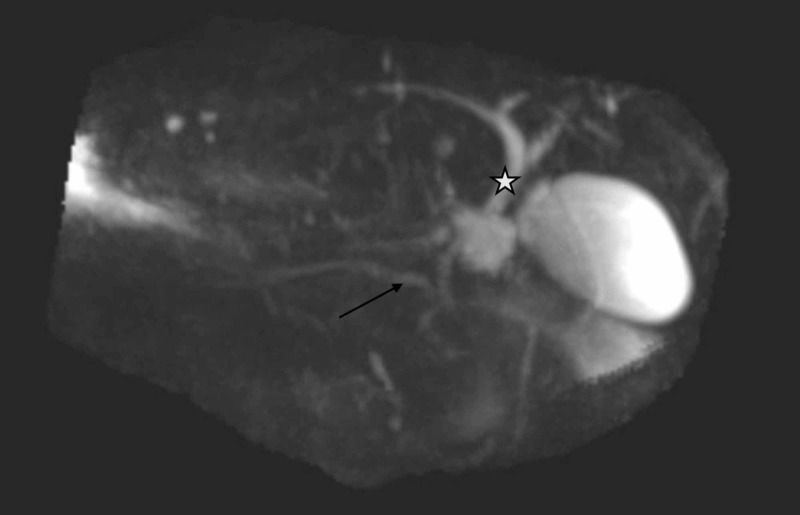
Magnetic resonance cholangiopancreatography showing normal anatomy of the main pancreatic duct (arrow) and normal common bile duct (star)

**Figure 3:**
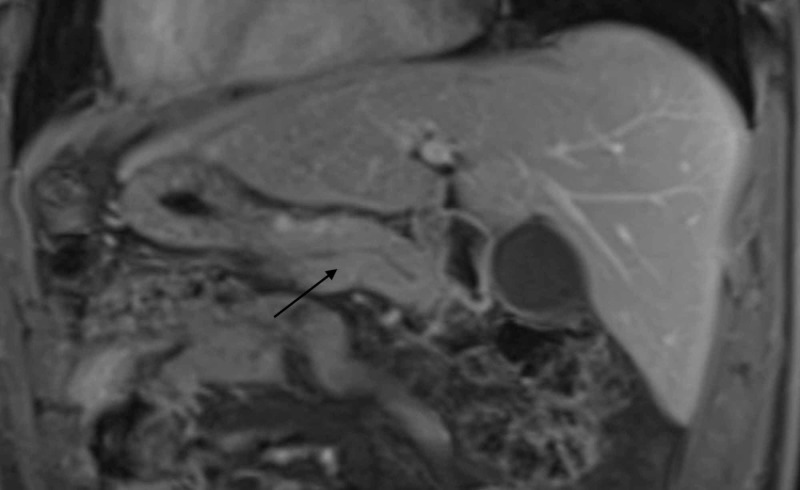
Normal pancreatic parenchyma on T1 imaging (arrow)

At his follow-up appointment, the patient disclosed that he started a new herbal supplement, B-sitosterol, for the first time, on the morning after his symptoms began. The patient only consumed two days of B-sitosterol from an over-the-counter preparation before his symptoms became intolerable, leading to hospitalization. Outpatient workup after discharge, including triglyceride level, amylase, lipase, and liver tests, remained within normal limits. An abdominal MRI and magnetic resonance cholangiopancreatography were obtained four months after his initial presentation to further exclude any pancreatic anomalies which showed interval resolution of pancreatitis and pancreatic fluid collections, normal anatomy of the pancreas as well as biliary and pancreatic ducts (Figures 2, 3).

There was no evidence of division. The patient was advised to avoid B-sitosterol and thus continued to remain asymptomatic. 

## Discussion

Drug-induced pancreatitis has been reported since the 1950s and each year the list of drugs associated with the condition continues to rise. Although a single cause is unknown, it has been proposed that drugs associated with acute pancreatitis act by direct toxicity, hypersensitivity, and other indirect mechanisms, such as hypercalcemia and intravascular thrombosis [3]. Drug-induced pancreatitis is classified based on the number of cases reported, demonstration of a consistent latency period, and reaction with drug rechallenge [4]. Establishing a diagnosis of drug-induced pancreatitis is difficult because most patients admitted to the hospital are on multiple medications and drug-induced pancreatitis is rarely associated with clinical or laboratory evidence and thus is a diagnosis of exclusion [3]. Based on the Naranjo scale, the causality of acute pancreatitis secondary to B-sitosterol in our patient was categorized as a probable event. Since this was the patient's first episode of pancreatitis and his symptoms improved with supportive treatment and removal of the drug, no further laboratory workup (i.e. thyroid-stimulating hormone or autoimmune) was done. Infectious etiology was ruled out in this case based on normal complete blood count, immunocompetent status of the patient, and the absence of other systemic signs.

Physicians should have a higher index of suspicion for drug-induced pancreatitis when clinically indicated in certain populations such as the elderly, HIV-positive patients, cancer patients, and patients receiving immunomodulating agents [5]. Saw palmetto, a phytotherapeutic agent commonly marketed for the treatment of BPH, has been reported in the literature as a probable cause of drug-induced pancreatitis in at least three published case reports. Similar to our case, Bruminhent et al. describe consistent resolution of symptoms during brief hospitalization after stopping the offending agent (saw palmetto) and excluding all other causes of acute pancreatitis [6]. Like B-sitosterol, saw palmetto is often used in combination with other plant extracts for the treatment of BPH when purchased over the counter.

Multiple reports in the literature have shown a causal relationship between corticosteroid use and acute pancreatitis. A postmortem study by Carone and Liebow demonstrated histologic evidence of acute pancreatitis in 30% of patients treated with corticotropin or adrenal steroids. Only 4% of matched controls in the group showed evidence of focal pancreatitis [7]. Nelp suggested that prolonged or high doses of steroids favored the development of acute pancreatitis although the mechanism remains unclear [8]. Although chronic illnesses may have played a role, failure to identify the association between acute pancreatitis and steroids may be responsible for the high mortality as well as the sparsity of cases reported. Nelp suggested that the incidence of pancreatitis is underreported due to three reasons: the lack of recognition of steroids inducing pancreatitis, the strong association of abdominal pain during steroid therapy with peptic ulcer, and the ability of steroids to mask the symptoms of pancreatitis [8].

B-sitosterol is a phytopharmacological drug containing mixtures of phytosterols and other sterols commonly used in the treatment of symptomatic BPH and hypercholesterolemia [9]. Phytosterols are naturally occurring plant sterols structurally similar to cholesterol obtained primarily through diet and supplementation [10]. In a study done by Racette et al, it was found that phytosterols in moderate (459 mg/day) and high (2,059 mg/day) doses significantly enhanced the excretion of biliary and dietary cholesterol and reduced the efficiency of intestinal cholesterol absorption relative to a phytosterol-deficient diet [11]. B-sitosterol is the main sterol in the Western diet. Natural sources of phytosterols include vegetable oil, seeds, cereals, nuts, whole grains, and plant oils, as well as b-sitosterol enriched margarines which are used in many cholesterol-lowering diets [12]. Through many pharmacologic studies, B-sitosterol has been deemed likely safe when taken by mouth in recommended doses for up to six months for BPH or for cholesterol-lowering effects. Because phytosterols occur in such small quantity in pure plant material, they are difficult to obtain in pure form and economically not profitable to synthesize on their own. When purchased commercially, in this case to help with BPH, B-sitosterol is often found in combination with other phytosterols [13]. Because this herbal supplement is not formally regulated, determining its specific toxicity and adverse effects becomes difficult. Although this plant-derived drug has been used for many years, the exact effectiveness and mode of action have not been determined. It is thought that B-sitosterol may be related to cholesterol metabolism or anti-inflammatory effects [10]. A Cochran review suggests that oral B-sitosterol is generally well tolerated with only mild adverse effects that are comparable in frequency to placebo. Gastrointestinal side effects, including abdominal pain, bloating, nausea, and constipation, were the most common side effects, occurring in 1.6% of men taking B-sitosterol compared to the placebo group [14]. Impotence was also reported in 0.5% of men taking B-sitosterol. 

## Conclusions

This is the first reported case of drug-induced pancreatitis from B-sitosterol, a common phytosterol found in many over-the-counter supplements worldwide. Our case demonstrates consistent resolution of symptoms and pancreatic enzymes along with normal imaging following discontinuation of the offending agent making this the first probable case reported in the literature. While drug-induced pancreatitis from corticosteroids and herbal supplement, saw palmetto, have been well described in the medical literature, the exact mechanism continues to remain unclear. By extension, the mechanism for how phytosterols can induce pancreatitis is even more obscure. With B-sitosterol being so widespread in the Western diet and in alternative medicine, it is important for consumers to understand the risks of taking supplements that are not formally regulated with obscure biological impacts. Nonetheless, with the rising use of herbal supplements which contain a vast variety of phytosterols, it is essential for healthcare professionals to obtain an accurate medication history and be aware of such serious adverse effects.
